# Forward Head Angle and Shoulder Angle in Relation to Stabilometry in Children with Pectus Excavatum Included in an Exercise Program

**DOI:** 10.3390/children13050664

**Published:** 2026-05-09

**Authors:** Marius Zoltan Rezumeș, Liliana Catan, Elena Constanta Amaricai, Ada Maria Codreanu, Andreea Ancuța Vataman, Vlad Laurentiu David

**Affiliations:** 1 Doctoral School, “Victor Babes” University of Medicine and Pharmacy, 300041 Timișoara, Romania; marius.rezumes@umft.ro (M.Z.R.); ada.codreanu@umft.ro (A.M.C.); andreea.vataman@umft.ro (A.A.V.); 2 Research Center for Assessment of Human Motion, Functionality and Disability, “Victor Babes” University of Medicine and Pharmacy, 300041 Timișoara, Romania; amaricai.elena@umft.ro; 3 Department of Rehabilitation, Physical Medicine and Rheumatology, Faculty of Medicine, “Victor Babes” University of Medicine and Pharmacy, 300041 Timișoara, Romania; 4 Department of Medicine, Faculty of Medicine, “Vasile Goldiș” Western University, 310025 Arad, Romania; 5 Department of Pediatric Surgery and Orthopedics, “Victor Babes” University of Medicine and Pharmacy, 300041 Timișoara, Romania; david.vlad@umft.ro; 6 Department of Pediatric Surgery and Orthopedics, Emergency Hospital for Children “Louis Țurcanu” Timișoara, 300011 Timișoara, Romania

**Keywords:** pectus excavatum, pediatric postural disorders, postural balance, exercise program, rehabilitation, stabilometry, sagittal alignment, postural control

## Abstract

**Highlights:**

**What are the main findings?**

- Pectus excavatum in children is associated with altered sagittal postural alignment and balance control.
- A structured exercise program is associated with improvements in head and shoulder alignment and postural stability.
- Changes in scapular alignment may be related to improvements in stabilometric parameters.

**What are the implications of the main findings?**

- Exercise-based rehabilitation may be considered in the conservative management of children with pectus excavatum.
- Combined postural and stabilometric assessment can support objective evaluation of treatment outcomes.
- Targeting thoracic mobility, breathing, and trunk muscle function may improve postural control.
- Early physiotherapy intervention may help reduce compensatory postural adaptations.
- These findings support the integration of physical therapy into pediatric orthopedic care.

**Abstract:**

**Background**: Pectus excavatum (PE) is the most common anterior chest wall deformity in children and adolescents. It may lead to postural adaptations of the trunk and spine and can influence the distribution of the center of gravity. **Methods**: A total of 35 patients with PE, with a Haller index < 3.25, aged 5–17 years, followed a structured exercise program including postural correction exercises, thoracic mobility exercises, breathing retraining, and trunk extensor strengthening for three months after proper instruction by a specialist. Patients were assessed before and after the intervention. Postural alignment was evaluated laterally (right and left) using the GaitON Posture Analysis System, and static balance was assessed using the PoDATA 2.0 stabilometric platform (Chinesport, Italy), which analyzes plantar pressure distribution and center of pressure (COP) displacement during orthostatic stance. Statistical analysis was performed using paired *t*-tests and Pearson correlation coefficients. **Results**: Stabilometric analysis demonstrated a reduction in COP trajectory length, confidence ellipse area, and maximum velocity, indicating improved postural control and reduced sway. Postural analysis revealed statistically significant improvements in head and shoulder girdle alignment. Correlations suggest a potential relationship between segmental alignment and stabilometric parameters and a possible reduction in thoracic hyperkyphosis associated with PE. **Conclusions**: Postural and stabilometric assessment in PE highlights changes in the analyzed parameters and suggest that a structured exercise program may be associated with improvements in biomechanical function and neuromuscular control. These methods can be integrated into conservative management and therapeutic strategies.

## 1. Introduction

Pectus excavatum is the most common congenital deformity of the anterior chest wall, characterized by a posterior depression of the sternum and adjacent costal cartilages. The severity of the deformity may vary considerably, from mild forms with predominantly esthetic impact to more pronounced forms that can influence respiratory function, exercise capacity, and patients’ quality of life [1,2]. The condition is usually identified in childhood; however, the deformity tends to become more evident during adolescence, particularly during periods of rapid musculoskeletal growth [3]. From an epidemiological perspective, pectus excavatum occurs more frequently in boys and represents one of the most common structural abnormalities of the chest wall encountered in pediatric practice [4].

In addition to the anatomical changes in the thorax, this deformity can lead to postural adaptations at the level of the trunk and spine. Children diagnosed with pectus excavatum frequently present postural patterns characterized by increased thoracic kyphosis, anterior projection of the shoulders, and alterations in scapular girdle positioning [5,6]. These adaptations can generate muscular imbalances between the anterior and posterior trunk musculature, influencing segmental stability and postural control. During childhood and adolescence, when the locomotor system undergoes active development, these changes can affect balance maintenance mechanisms and movement coordination [7].

Structural changes in the thorax can influence the distribution of the center of gravity and postural control strategies, leading to adaptations in trunk stabilization mechanisms. In this context, the assessment of postural stability provides important information regarding how the body compensates for biomechanical imbalances associated with chest wall deformities [8,9].

The management of pectus excavatum includes both surgical options and conservative approaches. Surgical interventions are usually recommended in severe cases or when the deformity produces significant functional limitations [10]. However, many children present mild or moderate forms that do not require or are not eligible for surgical correction. For these patients, exercise programs based on physiotherapy and postural re-education represent an important therapeutic option and aim to improve chest mobility, optimize respiratory mechanics, and correct muscular imbalances [11].

Exercise programs frequently include postural correction exercises, thoracic mobilization techniques, breathing exercises, and muscle strengthening components. These exercise programs aim to improve postural alignment, as well as neuromuscular control and overall musculoskeletal function [12].

In recent years, the objective assessment of postural stability has been increasingly used in rehabilitation studies through stabilometric analysis. These methods allow the quantification of center of pressure displacement during orthostatic stance and provide relevant information about balance control strategies. Stabilometric analysis represents a useful tool for monitoring neuromuscular adaptations and for evaluating the effectiveness of therapeutic programs [13].

Although previous studies have investigated postural alterations and balance in children with pectus excavatum, the relationship between segmental postural alignment—particularly at the level of the head and shoulder girdle—and stabilometric parameters remains insufficiently explored. Moreover, the role of sensorimotor control and postural regulation mechanisms in this context is not fully understood. Therefore, further research is needed to better clarify these interactions and their response to conservative therapeutic interventions.

The aim of the present study was to evaluate changes in postural stability and balance in children aged 5 to 17 years diagnosed with PE, following a three-month exercise program.

## 2. Materials and Methods

We conducted a prospective single group interventional study without a control group in 2025, within the Department of Medical Rehabilitation, Orthopedics Unit, at the Emergency Clinical Hospital for Children “Louis Țurcanu” Timișoara. The research protocol was approved by the Ethics Committee of the University of Medicine and Pharmacy “Victor Babeș” Timișoara (No. 12/10.02.2025), and the study was carried out in accordance with current ethical principles and regulations.

Written informed consent was obtained from the parents or legal guardians of the participants after providing clear information regarding the purpose and stages of the study. No personal identifying data were included in the study. Participants also received an informational leaflet and were informed about their right to withdraw from the study at any time.

### 2.1. Participants

A total of 45 patients diagnosed with pectus excavatum (PE) were included in this study. Participants were aged 5 to 17 years (mean age 11.23 ± 3.99), with a predominance of males. The inclusion criteria were: diagnosis of pectus excavatum in children or adolescents under 18 years of age, clinical presence of a mild anterior chest wall depression, confirmation by computed tomography (CT) for Haller index assessment, and willingness to participate in the required evaluations and to follow the exercise program for three months.

Patients older than 18 years (n = 3), those who had undergone surgery within the previous three months (n = 1), and those who did not agree to follow the daily exercise program (n = 2) were excluded. Participants with associated conditions that could influence the study outcomes were also excluded, including neurological disorders such as epilepsy (n = 1), cervical hyperlordosis (n = 2), or reduced physiological spinal curvature (n = 1). Following the application of these criteria, the final sample consisted of 35 participants.

For each participant, baseline values before the exercise program and post-intervention values after the exercise program were recorded for both stabilometric and postural parameters. Assessments were performed using the PoDATA 2.0 stabilometric platform and the GaitON Posture Analysis System.

In the stabilometric analysis, three main parameters were evaluated: center of pressure trajectory length (length of curve), confidence ellipse area (confidence ellipse area), and maximum center of pressure speed (maximum speed), which are commonly used indicators of postural stability.

Postural analysis was performed in the sagittal plane from right and left lateral views. Two main parameters were calculated: forward head angle (forward head angle) and shoulder angle (shoulder angle), used to assess head alignment and scapular girdle positioning.

#### 2.1.1. Sample Size Calculation

The sample size was calculated using G*Power software (version 3.1.9.7, Universität Kiel, Kiel, Germany). The effect size was set at 0.5, with a statistical significance level of α = 0.05 and a statistical power of 0.80. Based on these parameters, the minimum required sample size was 35 participants. The calculation was performed using a paired samples *t*-test for dependent measurements (pre–post design), consistent with the statistical methods applied in the analysis.

Intragroup analyses evaluated changes over time within the same group of participants. The interpretation of effect size followed the conventions proposed by Jacob Cohen for large effects [14]. The sample size is comparable to that reported by Zielinski (35 participants) in studies with similar statistical parameters [15].

#### 2.1.2. Objectives of the Study

The present study aimed to investigate the effects of a three-month physiotherapy exercise program on postural stability and postural alignment in children and adolescents diagnosed with pectus excavatum. Specifically, the study evaluated changes in stabilometric parameters, including the length of the center of pressure trajectory, confidence ellipse area, and maximum velocity, in order to quantify static balance. Additionally, postural alignment in the sagittal plane was assessed through the forward head angle and shoulder angle using a digital posture analysis system. Furthermore, the study explored the relationship between postural and stabilometric parameters to better understand the biomechanical and neuromuscular adaptations associated with pectus excavatum and their response to conservative therapeutic intervention.

### 2.2. Exercise Program

Within the study, participants followed a rehabilitation exercise program over a period of three months, between the baseline assessment (day 0) and the final assessment. The program was initiated on day 1, immediately after baseline evaluation, and was performed individually by each participant, including 10 exercises.

During the first 10 days, participants were instructed in the correct execution of the exercises under the supervision of a specialist, in daily 60 min sessions. Subsequently, the program was continued at home, with each exercise performed daily in 3 sets of 10 repetitions. Adherence to the program was monitored by parents through activity recording in a logbook.

**Figure 1:**
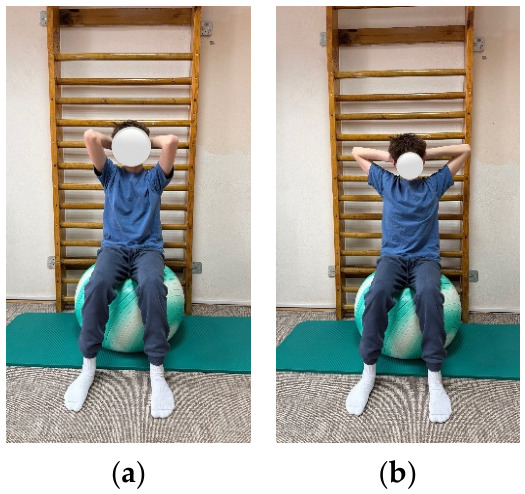
Exemplification of exercise: (**a**) initial position, (**b**) final position.

**Figure 2:**
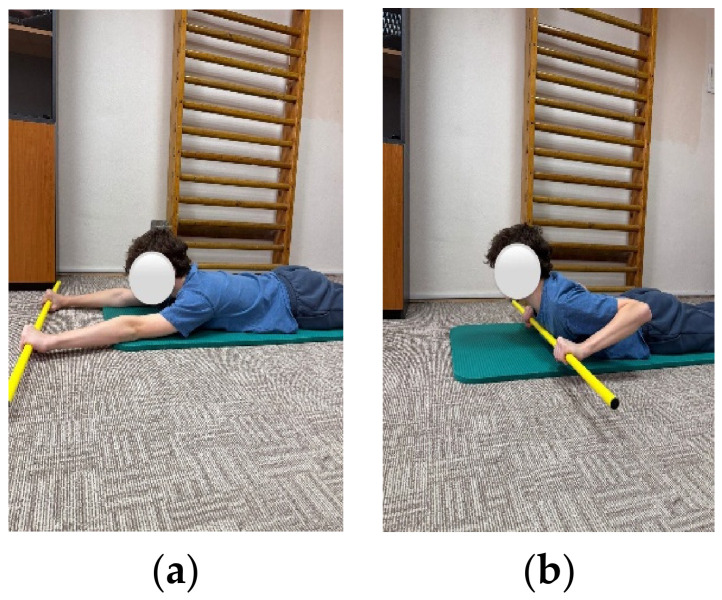
Exemplification of exercise: (**a**) initial position, (**b**) final position.

**Figure 3:**
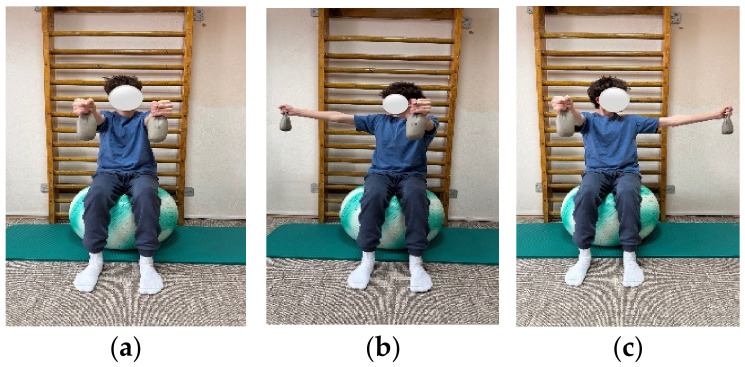
Exemplification of exercise: (**a**) initial position, (**b**) final position to the right, (**c**) final position to the left.

The objectives of the exercise program were to improve spinal mobility, correct posture, restore muscular balance, correct axial deviations, and retrain breathing. The program included exercises aimed at increasing spinal and thoracic mobility, as well as exercises for strengthening the trunk extensor muscles. These targeted strengthening of the back musculature through concentric training and stretching of the anterior trunk musculature. In addition, breathing retraining exercises and exercises to improve postural stability were included (Figure 1, Figure 2 and Figure 3).

The exercise program was progressively adapted based on each participant’s tolerance and ability, with gradual increases in exercise complexity and control over the three-month period. The intensity of the exercises was moderate and focused on controlled execution, correct posture, and breathing coordination rather than maximal effort. During the home-based phase, correct execution of the exercises could not be continuously supervised, which may have introduced variability in performance. Adherence to the program was monitored through parental supervision and activity logs; however, objective verification of compliance was limited [16].

### 2.3. Methods

The assessments included analysis of postural stability and evaluation of postural alignment in the sagittal plane. To reduce inter-rater variability, all evaluations were performed by the same physiotherapist experienced in postural and stabilometric analysis.

Static balance was assessed using the PoDATA 2.0 stabilometric platform (Chinesport, Udine, Italy). This system allows for the analysis of plantar pressure distribution and center of pressure (COP) displacement during orthostatic stance [17]. We assessed the participants with eyes open using one trial, according to this study protocol [16].

**Figure 4:**
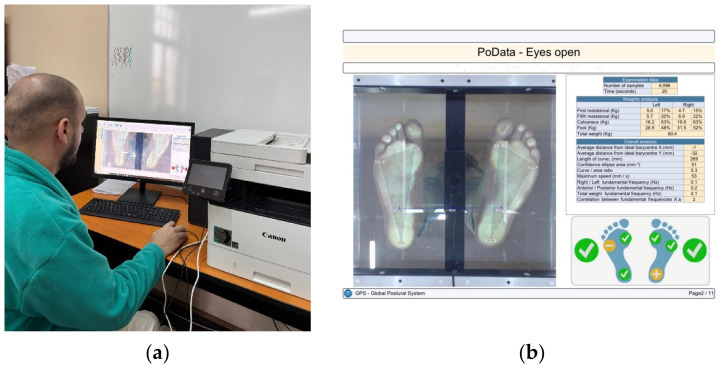
PoDATA technical setup: (**a**) computer interface and software, (**b**) exported report.

The platform is equipped with pressure sensors that record body weight distribution at the main support points of the foot, specifically at the first and fifth metatarsal heads and the heel. Based on these data, the dedicated software calculates stabilometric parameters such as center of pressure trajectory length, confidence ellipse area, and center of pressure speed, which are commonly used indicators in postural control analysis (Figure 4).

Before testing, participants were asked to remove their footwear to allow direct contact with the platform surface. After device calibration, each participant was positioned on the platform in an upright stance, with gaze directed forward and upper limbs relaxed alongside the body.

Postural alignment was assessed using the GaitON Posture Analysis System, a digital posture analysis system. This system allows static postural assessment based on images captured in the frontal and sagittal planes, providing information on segmental deviations from ideal biomechanical axes [18].

**Figure 5:**
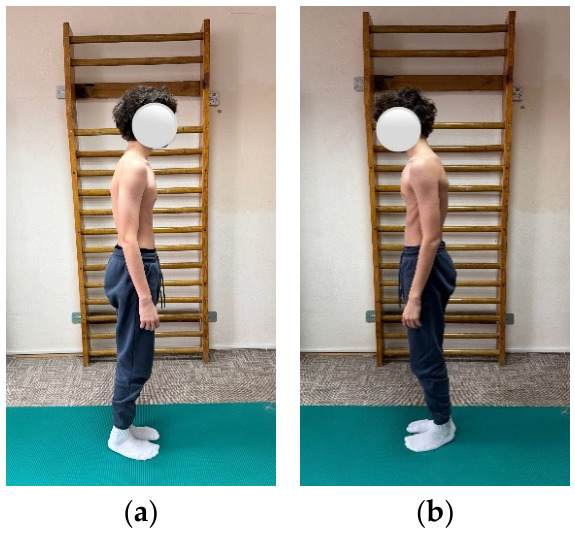
Patient positioning for postural assessment (**a**) right (**b**) left.

Postural analysis was performed from two perspectives: right lateral (Figure 5a) and left lateral (Figure 5b). To facilitate accurate identification of anatomical landmarks and proper alignment analysis, markers were placed on specific anatomical reference points (Figure 5). In the lateral view, on both the right and left sides, markers were positioned at the level of the C7 spinous process, the center of the humeral head, the greater trochanter, the lateral femoral epicondyle, and the lateral malleolus.

**Figure 6:**
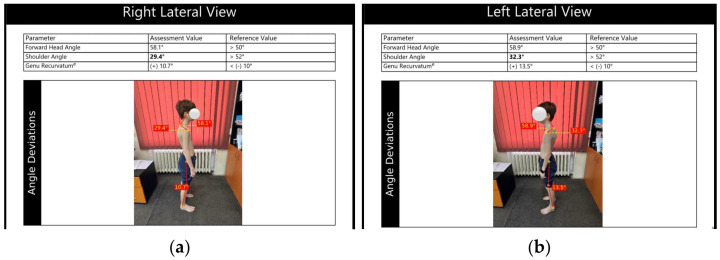
GaitON report: (**a**) right lateral view (**b**) left lateral view.

Based on these anatomical landmarks, the system automatically calculates specific postural parameters, including forward head angle and shoulder angle, used to assess head alignment and scapular girdle positioning in the sagittal plane. These measurements allow the identification of postural deviations commonly associated with biomechanical alterations observed in patients with chest wall deformities (Figure 6) [19,20].

### 2.4. Statistical Analysis

Statistical analysis was performed using MedCalc software (version 23.2.1; MedCalc Software Ltd., Ostend, Belgium). For each participant, baseline (pre-intervention) and post-intervention values were collected for the analyzed stabilometric and postural parameters. Data normality was assessed using the Shapiro–Wilk test prior to analysis. As the data followed a normal distribution, parametric tests were applied. Comparisons between baseline and post-intervention values were performed using the paired *t*-test.

For postural analysis, differences between pre- and post-intervention values were evaluated separately for the right and left lateral views. The analyzed parameters included forward head angle and shoulder angle.

To assess the relationship between postural and stabilometric parameters, correlation analysis was performed using the Pearson correlation coefficient. Effect sizes (Cohen’s d) were calculated to assess the magnitude of change, and 95% confidence intervals (CIs) were reported to improve the interpretability of the results.

Data are presented as mean ± standard deviation (SD). A *p*-value < 0.05 was considered statistically significant.

## 3. Results

In this section, the results of the data analysis obtained from stabilometric and postural assessments performed before and after the exercise program are presented. Results are reported as mean ± standard deviation, and the statistical significance of the observed differences was evaluated using appropriate statistical tests.

A total of 35 participants were included in the analysis, of whom 28 (80.0%) were male and 7 (20.0%) were female. The anthropometric characteristics of the participants are presented in Table 1.

The mean age of the participants was 11.23 ± 3.99 years, mean body weight was 43.14 ± 18.60 kg, and mean height was 151.86 ± 21.70 cm. The mean body mass index (BMI) was 17.77 ± 3.96 kg/m^2^. These values suggest a relatively homogeneous group in terms of age, sex distribution, and anthropometric characteristics.

### 3.1. Stabilometric Analysis

Stabilometric analysis demonstrated statistically significant reductions in postural sway parameters following the intervention (Table 2). Center of pressure trajectory length decreased from 531.06 ± 185.44 mm to 423.23 ± 166.91 mm (*p* < 0.001), with a medium effect size (Cohen’s d = 0.61). Confidence ellipse area and maximum center of pressure speed also showed statistically significant reductions after the intervention (all *p* < 0.001), with effect sizes of 0.44 and 0.16, respectively.

The reduction in center of pressure trajectory length, confidence ellipse area, and maximum speed is associated with improved postural control and reduced sway during orthostatic stance. In patients with pectus excavatum, these changes suggest that the exercise program may be associated with improved postural stability and more efficient neuromuscular adaptation involved in balance maintenance.

### 3.2. Postural Analysis

Postural analysis demonstrated significant improvements in head and scapular girdle alignment following the exercise program (Table 3).

The forward head angle increased significantly in both the right lateral view (51.44 ± 9.61° vs. 56.19 ± 7.74°, *p* < 0.001) and the left lateral view (50.88 ± 7.52° vs. 55.64 ± 6.09°, *p* < 0.001), with moderate effect sizes (Cohen’s d = 0.54 and 0.69, respectively).

Similarly, the shoulder angle showed significant increases after the intervention in both analyzed planes (right: 41.19 ± 12.09° vs. 49.64 ± 9.51°, *p* < 0.001; left: 41.42 ± 12.78° vs. 49.69 ± 10.18°, *p* < 0.001), with large effect sizes (Cohen’s d = 0.78 and 0.72) suggesting improved postural alignment following the exercise program.

The increase in forward head angle and shoulder angle is associated with improved head and scapular girdle alignment in the sagittal plane. In patients with pectus excavatum, sternal deformity frequently leads to biomechanical adaptations of the thorax and spine, including increased thoracic kyphosis, anterior head positioning, and shoulder protraction.

Improved head–thorax–scapular girdle alignment may be associated with a reduction in these compensatory postural adaptations. The observed findings may reflect changes in the sternum–thorax–scapula–head biomechanical chain and a potential reduction in the tendency toward thoracic hyperkyphosis commonly associated with pectus excavatum.

To evaluate the relationship between postural and stabilometric changes, correlation analysis was performed between Δ values (post-exercise program minus pre-exercise program) of the analyzed parameters using the Pearson correlation coefficient, as presented in Table 4.

Pearson correlation coefficients (r) were calculated to assess the relationship between changes (Δ = post − pre) in postural and stabilometric parameters.

The results demonstrated a moderate and statistically significant correlation between changes in the right shoulder angle and the stabilometric ellipse area (r = −0.47, *p* = 0.004). This association suggests a potential relationship between improved right scapular girdle alignment and reduced center of pressure oscillations. From a clinical perspective, this finding suggests that shoulder position may be associated with improved postural stability.

A significant negative correlation was also observed between changes in the right shoulder angle and center of pressure trajectory length (r = −0.34, *p* = 0.044), suggesting a possible association between scapular alignment and postural control.

For the remaining parameters, the observed correlations were weak and did not reach statistical significance (*p* > 0.05).

Overall, these results support the hypothesis that improved postural alignment may influence postural control mechanisms and body stability. This aspect is particularly relevant in patients with pectus excavatum, where chest wall deformity is frequently associated with alterations in scapular girdle positioning and trunk biomechanics.

Accordingly, therapeutic interventions targeting postural correction and scapulothoracic muscle re-education may play an important role in improving body alignment, postural stability, and balance control.

## 4. Discussion

The primary aim of this study was to evaluate changes in postural stability and postural alignment in patients with pectus excavatum (PE) following a three-month exercise program.

Postural assessment was performed using the GaitON system, based on observations from clinical practice indicating that children and adolescents with PE frequently present postural disturbances affecting the spine and limbs. These findings are supported by studies reported in the literature [18,21] which emphasize that, in patients with sternal deformities, spinal evaluation should not be overlooked for the identification of postural deviations, followed by referral to rehabilitation centers for appropriate correction [5,21].

The findings of the present study are consistent with those reported by Mete et al., who investigated 22 patients with PE and assessed spinal alignment and mobility using the Spinal Mouse system. Their results showed reduced spinal mobility, impaired spinal alignment, and decreased spinal position sense in adolescents with PE [7].

Although the assessment methods differ between studies, the results are comparable and support the concept that, in patients with PE, sternal deformity may lead to biomechanical adaptations of the thorax and spine, commonly associated with increased thoracic kyphosis, anterior head positioning, and shoulder protraction.

A daily exercise program performed over three months had a positive effect on postural improvement in children and adolescents with mild to moderate PE, as demonstrated by the results of the present study. This concept is also supported by published research, including the study by Davi de Podestá Haje et al., in which participants performed specific exercises targeting the anterior chest musculature at least five times per week, with breathing control and prior instruction by a physiotherapist. Their findings demonstrated the effectiveness of exercise programs in the correction or partial correction of PE [22].

A literature review published in 2025 by Sonel T. and Genec A. reported that pectus excavatum (PE) is frequently associated with postural alterations, affecting not only the cervical and thoracic regions but the entire spine, and may be accompanied by reduced spinal mobility [23].

Another review by Janssen N. et al. (2024) highlighted that PE may be associated with various musculoskeletal conditions, including scoliosis and connective tissue disorders, as well as multiple genetic conditions [24].

Thoracic kyphosis and forward shoulder positioning are commonly reported clinical features in patients with PE, findings that are also consistent with the results of the present study. These observations support the importance of a comprehensive spinal assessment in children and adolescents presenting with thoracic deformities.

In addition, the assessment of postural stability in patients with PE may be useful for identifying balance impairments and guiding rehabilitation strategies. Exercise-based interventions have been reported to contribute to improvements in posture, spinal mobility, muscle balance, and respiratory function; however, these effects may vary depending on individual characteristics, and exercise programs should be tailored accordingly [21].

Regarding postural analysis in the present study, the results showed significant increases in forward head angle and shoulder angle values in both right and left lateral views. These changes indicate improved head and scapular girdle alignment in the sagittal plane and suggest a reduction in compensatory postural adaptations, as well as improved segmental alignment.

These observations are similar to those reported in the specialized literature. A relevant contribution in this context is a meta-analysis published in 2024 by Sepehri et al., including 22 studies with a total of 903 participants. It analyzed the impact of physical exercise interventions, including strength training, stretching, and shoulder exercises, on upper trunk abnormalities such as thoracic hyperkyphosis, anteriorly rounded shoulders, and forward head posture. The study demonstrated the effectiveness of these interventions in improving forward head angle, shoulder rounding, and thoracic kyphosis [25].

Kim et al. also concluded in their 2019 study that the craniovertebral angle and mean plantar pressure distribution improve as a result of controlled muscle activity in the neck and shoulder region induced by exercise sessions, highlighting the relationship between the forward head posture angle and the mean plantar pressure ratio [26].

The assessment of static balance using stabilometry (PoDATA) represents an additional approach in the evaluation of children with PE. A review of the literature did not identify studies reporting results based on this method in this population.

The stabilometric analysis performed in the 35 participants with PE showed that postural changes associated with this condition influence key parameters, including center of pressure trajectory length, confidence ellipse area, and maximum center of pressure speed. These findings are consistent with those reported by Nazli Elif Nacar et al., who observed that static balance in children with thoracic hyperkyphosis is affected, particularly in the absence of visual input, and that balance with eyes closed is associated with changes in trunk and lower limb muscle function [27].

However, other studies report different findings. For example, Hussein Youssef et al., in a study conducted on subjects with postural thoracic kyphosis, did not identify significant relationships between body balance and thoracic kyphosis [28].

Comparing post-intervention and baseline stabilometric parameters in children and adolescents with PE, a significant reduction was observed in center of pressure trajectory length, confidence ellipse area, and maximum speed. These findings indicate reduced postural sway and improved balance control during orthostatic stance.

A reduction in the amplitude of center of pressure displacement is commonly interpreted as an indicator of improved neuromuscular strategies involved in maintaining postural stability. This concept is supported by other authors, including Yun-Jin Park et al. [29] and Seoyon Yang et al. [30], who reported that combined exercise programs are effective in correcting spinal deviations and improving balance in adolescents with postural kyphosis.

The correlations identified between changes in postural and stabilometric parameters suggest a functional relationship between postural alignment and stability control. Improved head and scapular girdle alignment may influence thoracic mechanics and load distribution at the spinal level, contributing to improved postural control.

The results and observed correlations support the hypothesis that a three-month exercise program in children and adolescents with PE, focused on postural correction, may have beneficial effects on both segmental alignment and overall postural stability.

Due to the absence of a control group, causal relationships cannot be established, and the observed changes should be interpreted as associations. These findings may also be influenced by factors such as neuromuscular coordination, motor learning, or repeated testing effects.

### Study Limitations

The relatively small sample size and the absence of a control group limit the generalizability of the findings and restrict the ability to establish causal relationships between the intervention and the observed changes. The wide age range of participants (5–17 years) may introduce variability related to growth and developmental stages, which may have influenced the magnitude of the observed changes and should be considered when interpreting the results. In addition, thoracic kyphosis was not directly assessed, and the analysis focused on head and shoulder alignment in the sagittal plane.

Furthermore, the largely unsupervised nature of the home-based exercise program may have introduced variability in both execution and adherence. Future studies should include comprehensive assessments of spinal curvatures and larger, more homogeneous patient cohorts.

## 5. Conclusions

The findings suggest that a structured exercise program including postural correction, thoracic mobility, and breathing exercises may be associated with improvements in postural alignment and stabilometric parameters in patients with pectus excavatum. These results may suggest a potential role of individualized physiotherapy programs in the conservative management of this condition and should be interpreted as exploratory given the single group design.

The applied methods may be integrated into the conservative management of this condition and further controlled studies are needed.

## Figures and Tables

**Table 1:** Anthropometric data of the group shown.

Variable	Mean ± SD
Age (years)	11.23 ± 3.99
Weight (kg)	43.14 ± 18.60
Height (cm)	151.86 ± 21.70
BMI (kg/m^2^)	17.77 ± 3.96

cm: centimeter; kg: kilogram; BMI: body mass index; SD: standard deviation.

**Table 2:** Stabilometric parameters before and after the exercise program (n = 35).

Parameter	Before the Exercise Program (Mean ± SD)	After the Exercise Program (Mean ± SD)	95% CI	Effect Size (Cohen’s d)	*p*-Value
Length of curve (mm)	531.06 ± 185.44	423.23 ± 166.91	−140.48 to −75.18	0.61	**<0.001**
Confidence ellipse area (mm^2^)	215.89 ± 152.34	153.40 ± 127.94	−89.16 to −35.81	0.44	**<0.001**
Maximum speed (mm/s)	174.11 ± 196.55	143.74 ± 184.04	−46.41 to −14.34	0.16	**<0.001**

Values are expressed as mean ± standard deviation (SD). Statistical significance was assessed using the paired *t*-test. Effect size is reported as Cohen’s d. CI: confidence intervals. A *p*-value < 0.05 was considered statistically significant. *p*-value: probability value; SD: standard deviation; mm/s: millimeters per second; mm^2^: square millimeters; mm: millimeter.

**Table 3:** Postural parameters before and after the exercise program (n = 35).

View	Right Lateral View					Left Lateral View				
Parameter	Before (Mean ± SD)	After (Mean ± SD)	Effect Size	95% CI	*p*-Value	Before (Mean ± SD)	After (Mean ± SD)	Effect Size	95% CI	*p*-Value
Forward Head Angle (°)	51.44 ± 9.61	56.19 ± 7.74	0.54	3.53 to 5.98	<0.001	50.88 ± 7.52	55.64 ± 6.09	0.69	3.77 to 5.75	<0.001
Shoulder Angle (°)	41.19 ± 12.09	49.64 ± 9.51	0.78	6.68 to 10.21	<0.001	41.42 ± 12.78	49.69 ± 10.18	0.72	6.54 to 9.99	<0.001

Values are expressed as mean ± standard deviation (SD). Statistical significance was assessed using the paired *t*-test. A *p*-value < 0.05 was considered statistically significant. °: angle formed; SD: standard deviation; CI: confidence interval; Effect sizes are reported as Cohen’s d; *p*-value: probability value.

**Table 4:** Pearson correlation coefficients (r) between changes (Δ = post − pre) in stabilometric and postural parameters (n = 35).

Parameter	Δ Length of Curve	Δ Confidence Ellipse Area	Δ Maximum Speed
Δ Forward Head Angle—Left	r = − 0.03 (*p* = 0.840)	r = −0.07 (*p* = 0.700)	r = −0.04 (*p* = 0.799)
Δ Shoulder Angle—Left	r = 0.11 (*p* = 0.532)	r = −0.04 (*p* = 0.817)	r = −0.22 (*p* = 0.188)
Δ Forward Head Angle—Right	r = 0.12 (*p* = 0.486)	r = 0.08 (*p* = 0.638)	r = 0.02 (*p* = 0.884)
Δ Shoulder Angle—Right	r = **−0.34 (*p* = 0.044)**	r = **−0.47 (*p* = 0.004)**	r = −0.22 (*p* = 0.202)

**Note:** Δ represents the change score (post − pre). Values are expressed as Pearson correlation coefficients (r) with corresponding *p*-values. Statistically significant correlations are highlighted in bold.

## Data Availability

The data presented in this study are available on request from the corresponding author (L.C.) due to privacy.

## Abbreviations

The following abbreviations are used in this manuscript:

FHA	Forward head angle
SA	Shoulder angle
PE	Pectus excavatum
COP	Center of pressure
CT	Computerized tomography
SD	Standard deviation
BMI	Body mass index
*p*≈	*p* is approximately equal to
Δ	difference
°	angle formed
*p*-value	probability value
mm/s	millimeters per second
mm^2^	square millimeters
mm	millimeters
cm	centimeter
kg	kilogram
